# Long non-coding RNA RP4-694A7.2 Promotes Hepatocellular Carcinoma Cell Proliferation and Metastasis through the Regulation of PSAT1

**DOI:** 10.7150/jca.59348

**Published:** 2021-07-25

**Authors:** Yaoxin Fan, Lin Wang, Yang Ding, Qiuju Sheng, Chong Zhang, Yanwei Li, Chao Han, Bingchao Lu, Xiaoguang Dou

**Affiliations:** 1 Department of Infectious Diseases, Shengjing Hospital of China Medical University, No. 39, Huaxiang Road, Shenyang Liaoning province, China; 2 Key Laboratory of Viral hepatitis, Shengjing Hospital of China Medical University, No. 39, Huaxiang Road, Shenyang Liaoning province, China; 3 Department of Health Management, Shengjing Hospital of China Medical University, No. 39, Huaxiang Road, Shenyang Liaoning province, China

**Keywords:** lncRNA RP4-694A7.2, cell proliferation, invasion and migration, hepatocellular carcinoma

## Abstract

**Background:** Long noncoding RNAs (lncRNAs) have emerged as gene regulators in various cancers, including hepatocellular carcinoma (HCC). However, the biological roles and mechanisms of many lncRNAs in HCC tumorigenesis remain unknown.

**Aim:** To identify novel lncRNAs associated with proliferation and metastasis in HCC.

**Methods:** Expression profiles of lncRNAs were analyzed in HCC using two GSE datasets (GSE94660 and GSE104310). Functional studies were performed, including cell proliferation, colony formation, wound healing, and Transwell assays. Fluorescence in-situ hybridization (FISH), tandem mass tag (TMT) analyses, parallel reaction monitoring (PRM), and rescue assays were performed to evaluate the mechanisms underlying the effects of RP4-694A7.2.

**Results:** RP4-694A7.2 levels were higher in HCC tissues than in normal liver tissues in published GSE datasets and were elevated in HCC cell lines. Cell function assays revealed that RP4-694A7.2 promotes cell proliferation, invasion, and migration. Furthermore, RP4-694A7.2 was primarily found to be located in the cytoplasm by FISH assay. Then, TMT assay was performed to predict proteins associated with RP4-694A7.2, and 28 cytoplastic proteins were identified by PRM. Finally, phosphoserine aminotransferase 1 (PSAT1) was found to be regulated by RP4-694A7.2 to modulate growth and metastasis in HCC cells using a rescue assay.

**Conclusions:** These results suggested that RP4-694A7.2 promotes HCC cell proliferation and metastasis via PSAT1, providing a candidate therapeutic target for further research.

## Introduction

Hepatocellular carcinoma (HCC) is a leading cause of cancer-related deaths worldwide. Despite treatment advances, HCC mortality is still high 1,2. The lack of therapeutic strategies can be explained by the limited understanding of the mechanisms underlying HCC, which are complex diverse and vary among individuals 3. Therefore, studies of the underlying mechanism are expected to improve outcomes and decrease mortality in HCC.

Long non-coding RNAs (lncRNAs) are a class of non-coding RNAs more than 200 nucleotides in length 4. lncRNAs regulate gene expression at the epigenetic, transcriptional, and post-transcriptional levels and can promote or suppress cell signaling pathways in different types of cancer 5,6. Accumulating evidence has revealed the importance of lncRNAs in the development of HCC, including roles in proliferation, angiogenesis, metastasis, and genomic stability 7-13. Although several lncRNAs involved in the development of HCC have been identified, the precise functions of most lncRNAs have not been characterized. For example, lncRNA RP4-694A7.2 was previously described to be upregulated in some solid cancers compared to their respective normal tissues through bioinformatics analyses 14. This lncRNA was also overexpressed in highly proliferative stages of normal B cell differentiation 15. Although those studies showed that RP4-694A7.2 was associated with cancers, the function of the molecule in most cancers remains unclear.

In the present study, differentially expressed lncRNAs in two HCC-related GSE datasets (GSE94660 and GSE104310) were analyzed. The expression and localization of the candidate lncRNA RP4-694A7.2 were further assessed in HCC cells. Functional experiments suggested that RP4-694A7.2 promotes proliferation and metastasis in HCC. Phosphoserine aminotransferase 1 (PSAT1), an important factor in HCC, was regulated by PR4-694A7.2, as determined by tandem mass tags and parallel reaction monitoring. Our study suggested that RP4-694A7.2 could be a potential therapeutic target in HCC.

## Materials and Methods

### GEO data

GEO (Gene Expression Omnibus, http://www.ncbi.nlm.nih.gov/geo) is an international public database of microarray data, second-generation sequencing data, and other high-throughput sequencing data 16. Two GSE (series) HCC datasets (GSE94660 and GSE104310) were downloaded from the GEO.

### lncRNA expression profile analysis

lncRNA-Seq high-throughput sequencing and subsequent bioinformatics analyses were all performed by Cloud-Seq Biotech (Shanghai, China). Briefly, paired-end reads were subjected to a quality control threshold of Q30. After 3′ adaptor-trimming and low-quality read removal using cutadapt (v. 1.9.3), the high-quality trimmed reads were aligned to the human reference genome (UCSC hg19) using HISAT2 (v. 2.0.4). Next, HTSeq (v. 0.9.1) was used to obtain raw counts at the gene level (mRNA) and transcript level (lncRNA) as the expression profiles, and edgeR (v. 3.16.5) was used to normalize the counts and screen differentially expressed lncRNAs and mRNAs.

### Cell culture

LO_2_ and SK-Hep1cells were purchased from the Cell Bank, Type Culture Collection, Chinese Academy of Sciences (CBTCCCAS). All cells were incubated under standard culture conditions (37°C and 5% CO_2_).

### Quantitative real-time PCR (RT-PCR)

Total RNA was extracted from cells using TRIzol (Invitrogen, Carlsbad, CA, USA) following the manufacturer's instructions. Real-time PCR was conducted using the SYBR Green PCR Kit (Takara Biotechnology, Dalian, China) and the LightCycler 480 system following the manufacturer's instructions. *GAPDH* was used as a control. Relative expression levels were calculated using the 2^-ΔΔCt^ method. All primers are described in Table 1.

### LV-RP4-694A7.2-RNAi construction

For RP4-694A7.2 knockdown, interfering lncRNAs targeting RP4-694A7.2 were acquired from GeneChem (Shanghai, China). To construct LV-RP4-694A7.2-RNAi, SK-Hep1 cells suspended in DMEM were incubated in a 6-well plate. Next, cells were incubated with the lentivirus packaging vector (GV112). Fluorescence was captured using a fluorescence microscope (Olympus, Tokyo, Japan) at 72 h after lentiviral infection. The knockdown efficiency was examined by RT-PCR.

### Cell proliferation assay

SK-Hep1 cells were incubated in 96-well plates (2000 cells per well) under standard culture conditions. Cell viability was measured using Cell Counting Kit-8 (CCK-8; Dojindo, Kumamoto, Japan). The plates were detected using a Microplate Reader (Tecan Infinite, M2009PR) at 450 nm after 4 h of incubation.

### Colony formation assay

After transfection for 72 h, SK-Hep1 cells were incubated in 6-well plates (1000 cells per well). After culture for 2 weeks, colonies were dyed with Crystal Violet Staining Solution and evaluated.

### Wound-healing assay

Suspended SK-Hep1 cells were added to a 96-well plate at 5 × 10^5^/mL per well. A scratch tester was used to draw lines on the plates. Next, samples were washed with serum-free medium and added to serum-based medium. Images were obtained at 0, 24, and 32 h. Cell mobility (%) was calculated according to the following formula: (1 - scratch width/initial scratch width) × 100.

### Transwell assay

SK-Hep1 cells were suspended and incubated in 24-well plates (5 × 10^5^ cells per well). In the upper chamber, 100 µL of cell suspension was added. In the lower chamber, 600 µL of FBS culture medium was added. After 16 h of culture, the chambers were stained with Crystal Violet Staining Solution and evaluated.

### Fluorescence in-situ hybridization (FISH)

SK-Hep1 cells were fixed in paraformaldehyde (DEPC) for 20 min and washed three times with PBS. Proteinase K (20 µg/mL) was added to cover tissues, followed by incubation at 37°C for 8 min. Hybridization buffer was added to the specimen, followed by incubation at 37°C for 1 h. The probe hybridization solution was added and incubated in a humidity-controlled chamber for hybridization at 37°C overnight. Samples were incubated with DAPI for 8 min in the dark and mounted. Images were obtained using a positive fluorescence microscope.

### Tandem mass tag (TMT)

After samples were digested, they were labeled using TMT reagent according to the manufacturer's instructions (Thermo Fisher Scientific, Waltham, MA, USA). TMT-labeled peptides were fractionated by RP chromatography using the Agilent 1260 infinity II HPLC and evaluated by LC-MS/MS. A peptide and protein false discovery rate of 1% was enforced using a reverse database search strategy 17. Proteins with fold change > 1.2 and p-value (Student's *t*-test) < 0.05 were considered differentially expressed.

### Parallel reaction monitoring (PRM) analysis

To verify the accuracy of the TMT and LC-MS/MS analyses, PRM protein information was imported into Xcalibur for an analysis using PRM parameter settings. Peptides (1 µg) were collected from each sample for LC-PRM/MS, and chromatographic separation was performed using the Easy nLC 1200 chromatography system (Thermo Scientific) with a nanoliter flow rate after loading. The sample was subjected to gradient separation through a chromatography column (Thermo Fisher Scientific; Acclaim PepMap RSLC 50 µm × 15 cm, nano viper, P/N164943). Peptide fragmentation and targeted PRM mass spectrometry were performed using a Q-Exactive HF-X Mass Spectrometer (Thermo Scientific). Raw data obtained by mass spectrometry were analyzed using SpectroDive for PRM data.

MS raw data were processed using Proteome Discoverer (v. 2.1) and SpectroDive (v. 10.0). In Proteome Discoverer, the derived peak lists were searched using the MASCOT engine (Matrix Science, London, UK; version 2.6) against the UniProt *Homo sapiens* protein database (UP000005640, 20367 sequences, downloaded on February 26, 2020). In SpectroDive, the MS/MS table output file from MASCOT was used to create a spectral library.

### Statistical analysis

All data were analyzed using SPSS v. 22.0 (SPSS Inc., Chicago, IL, USA). Differences between control and experimental groups were evaluated by Student's *t*-tests. Data are reported as means ± standard deviation. Values of *p* < 0.05 were considered significant.

## Results

### RP4-694A7.2 was upregulated in HCC

**Figure 1:**
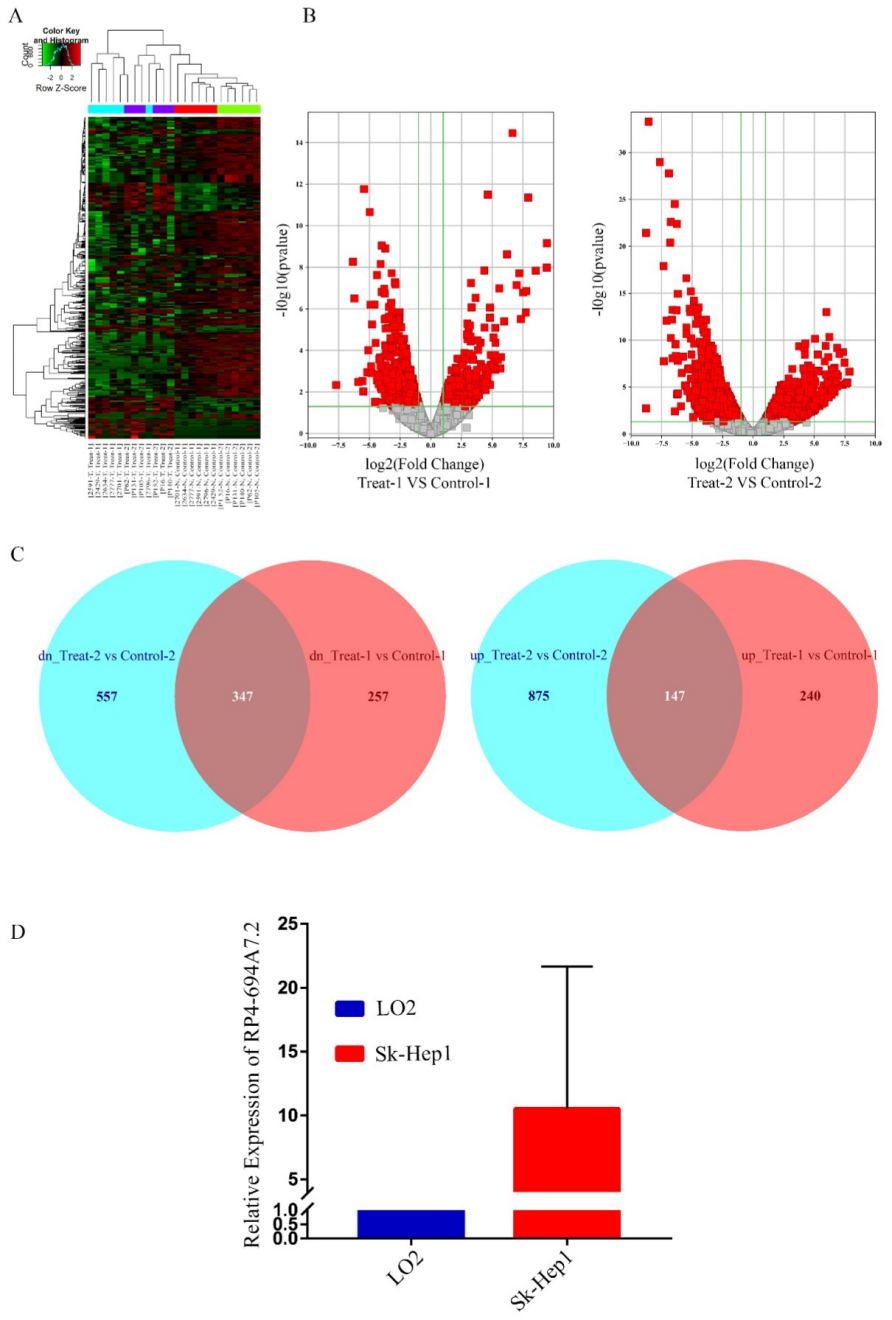
** RP4-694A7.2 levels were increased in HCC. A:** Dysregulated lncRNAs were screened by a bioinformatics approach using the GSE94660 and GSE104310 datasets. **B:** Volcano map of differentially expressed lncRNAs**. C:** Venn diagram showing the intersection between lncRNAs in the GSE94660 and GSE104310 datasets. **D:** RP4-694A7.2 was examined by qRT-PCR in HCC (SK-Hep-1) and LO_2_ cell lines (*p* < 0.05).

To screen unique lncRNAs related to HCC, two GSE datasets (GSE94660 and GSE104310) were downloaded from the GEO for bioinformatics analyses. In total, 1409 lncRNAs were upregulated while 1508 lncRNAs were downregulated in HCC. Heat maps for the GSE94660 and GSE104310 datasets are shown in Figure 1A, and a volcano map of differentially expressed lncRNAs is shown in Figure 1B. In total, 387 lncRNAs were upregulated and 604 were downregulated in GSE104310, while 1022 lncRNAs were upregulated and 904 were downregulated in GSE94660. A Venn diagram was generated to visually evaluate the overlap between lncRNAs. We found 347 upregulated and 147 downregulated lncRNAs (Figure 1C).

We focused on upregulated lncRNAs, which are more suitable than downregulated lncRNAs as diagnostic biomarkers or therapeutic targets 18. The top 50 upregulated lncRNAs in GSE94600 and GSE104310 were analyzed. RP4-694A7.2 was a highly expression lncRNA and was therefore further evaluated in SK-Hep1 cells by qRT-PCR. Compared with levels in LO_2_ cells, RP4-694A7.2 was significantly upregulated in SK-Hep1 cells (Figure 1D). Accordingly, RP4-694A7.2 might play a role in HCC.

### Knockdown of RP4-694A7.2 inhibits HCC cell proliferation

**Figure 2:**
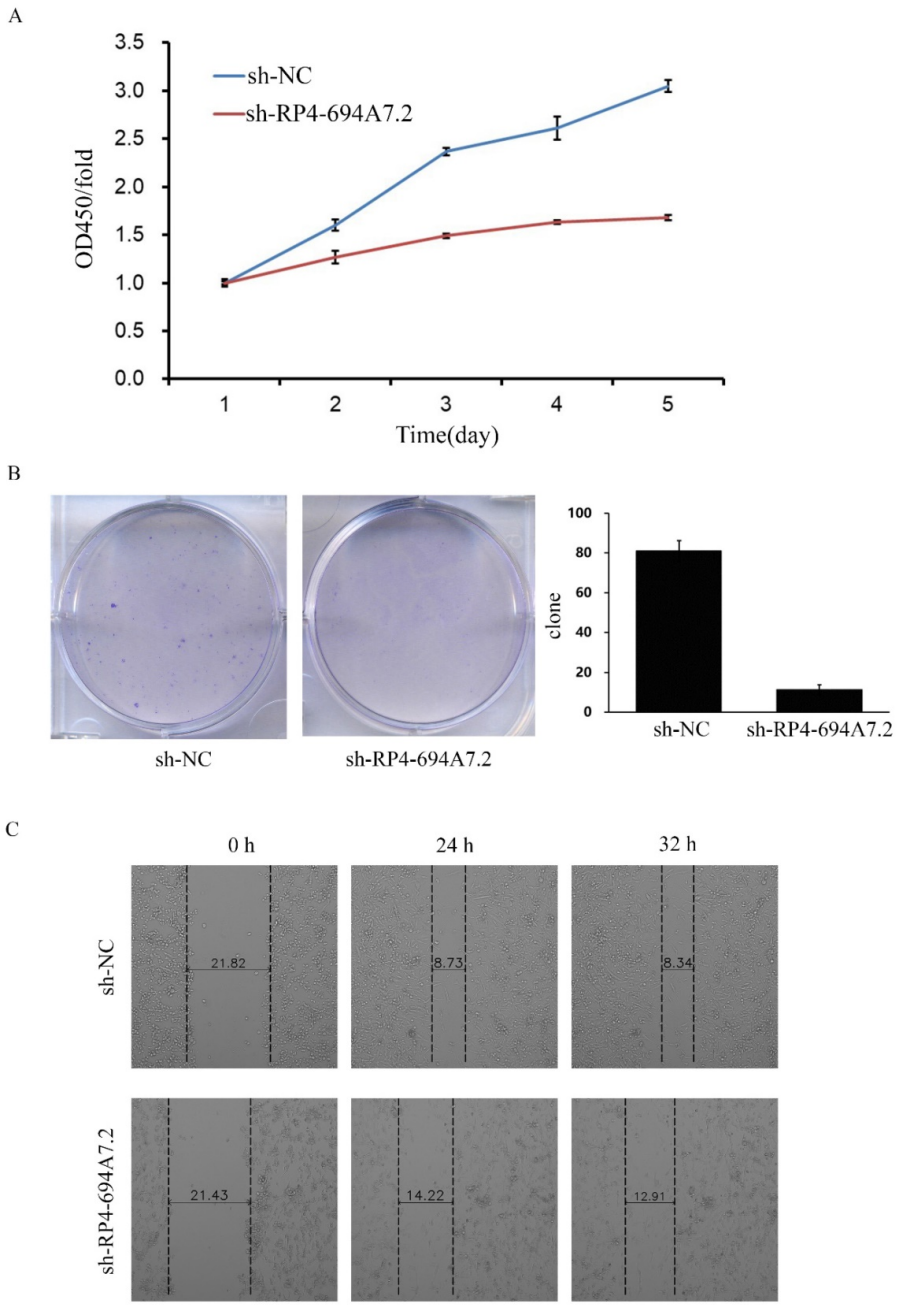

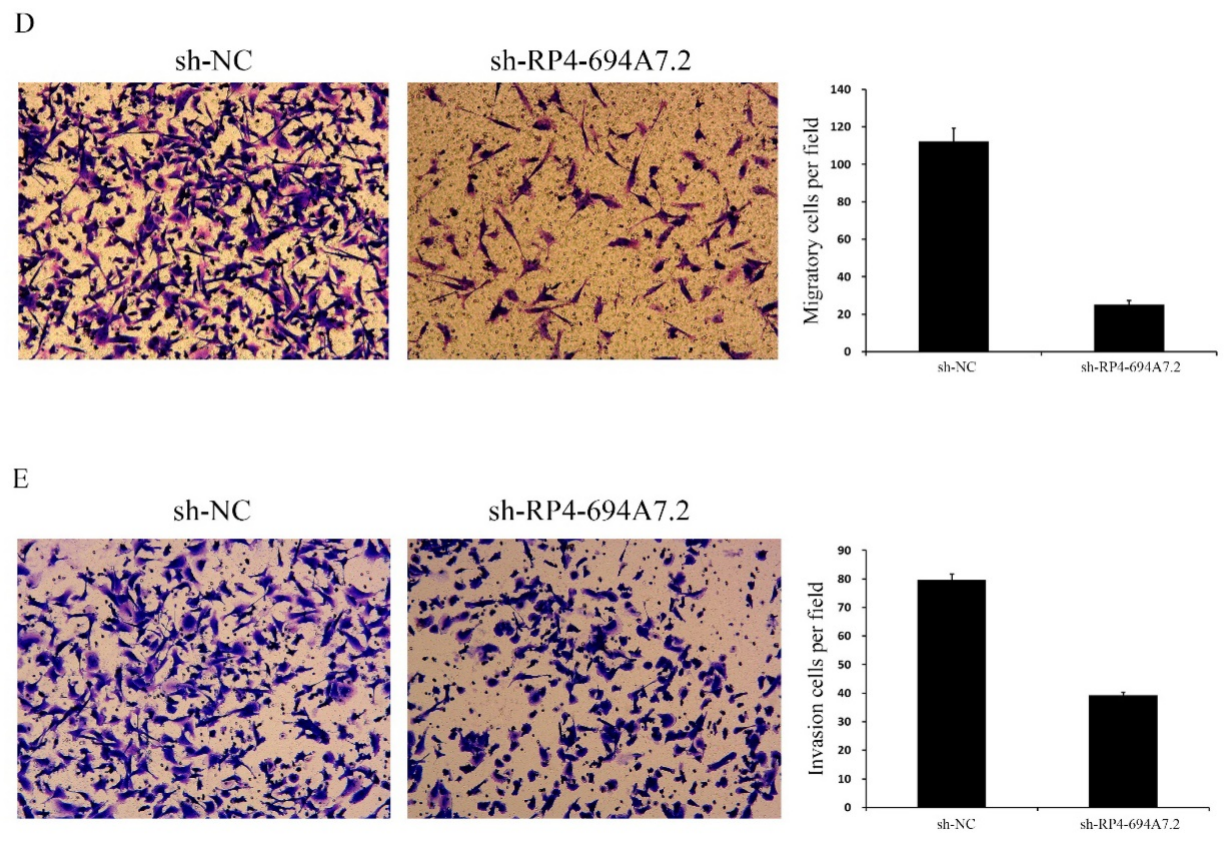
** Knockdown of RP4-694A7.2 inhibited HCC cell proliferation, migration, and invasion. A:** Knockdown of RP4-694A7.2 in HCC cells inhibited proliferation, as determined by a CCK-8 assay (*p* < 0.05). **B:** Knockdown of RP4-694A7.2 inhibited the formation of cell clones (*p* < 0.05).** C:** Knockdown of RP4-694A7.2 inhibited HCC cell migration, as determined by a wound-healing assay. **D:** Knockdown of RP4-694A7.2 inhibited the migration of HCC cells, as determined by a Transwell assay (*p* < 0.05). **E:** Knockdown of RP4-694A7.2 inhibited the invasion of HCC cells, as determined by a Transwell assay (*p* < 0.05). Data are expressed as means ± SD of three independent experiments.

To explore the function of RP4-694A7.2 in HCC proliferation, we knocked down RP4-694A7.2 in SK-Hep-1 cells and confirmed the knockdown efficiency by qRT-PCR. The RP4-694A7.2 level was markedly decreased in HCC cells after knockdown. According to a CCK-8 assay, RP4-694A7.2 knockdown remarkably reduced the rate of cell proliferation compared with that in the control group on days 4 and 5 in SK-Hep-1 cells (Figure 2A). The knockdown of RP4-694A7.2 also inhibited SK-Hep-1 cell proliferation in a colony formation assay (Figure 2B).

### Knockdown of RP4-694A7.2 inhibits HCC cell migration and invasion

We further evaluated the effects of RP4-694A7.2 knockdown on migration and invasion in HCC cells. According to a wound-healing assay, RP4-694A7.2 knockdown delayed wound healing (Figure 2C), suggesting reduced migration. Based on Transwell migration and invasion assays, the knockdown of RP4-694A7.2 significantly reduced migrating and invading cell counts (Figure 2D).

Taken together, RP4-694A7.2 knockdown could inhibit HCC cell migration and invasion.

### Screening proteins associated with RP4-694A7.2 in HCC cells

**Figure 3:**
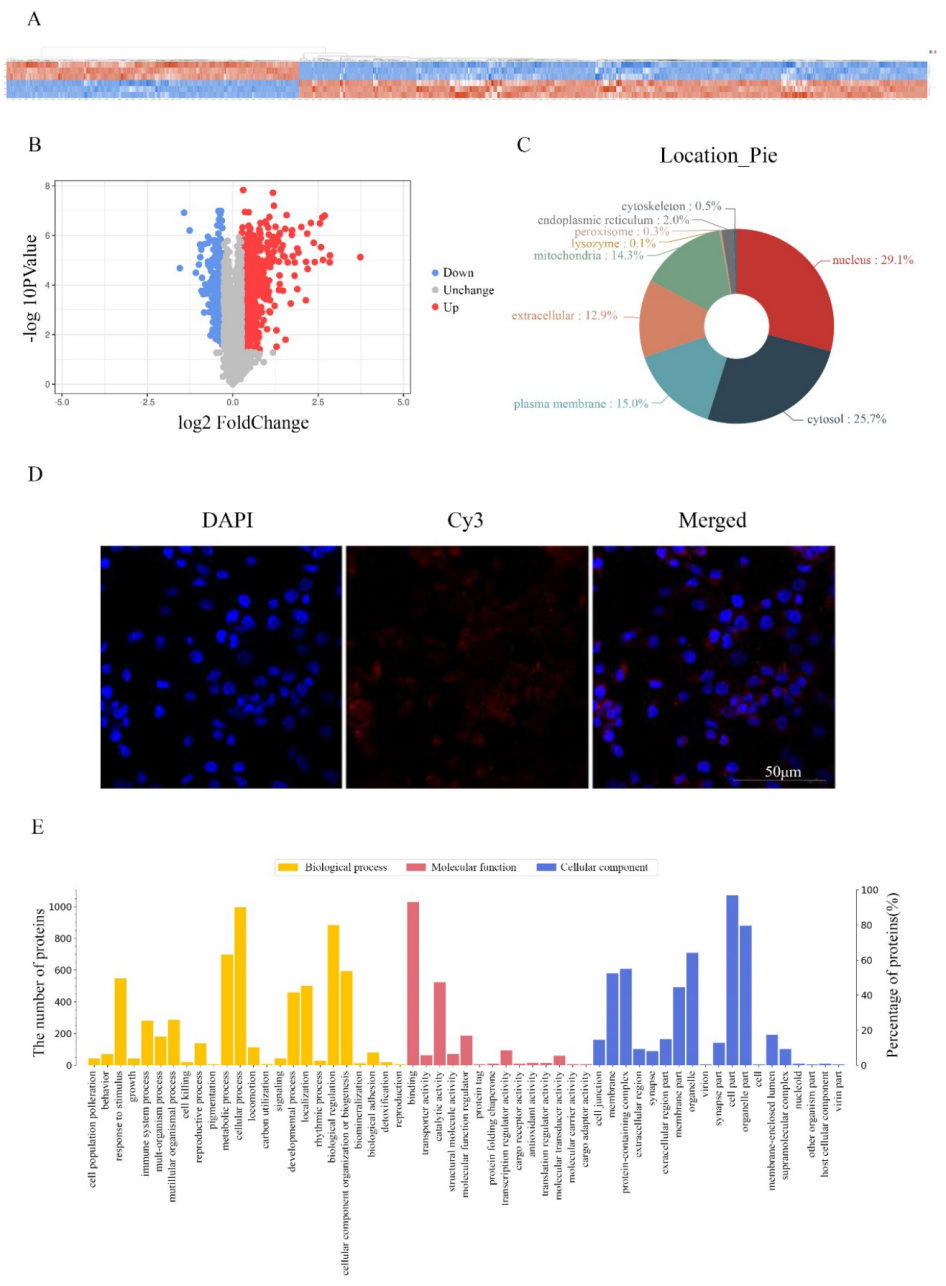

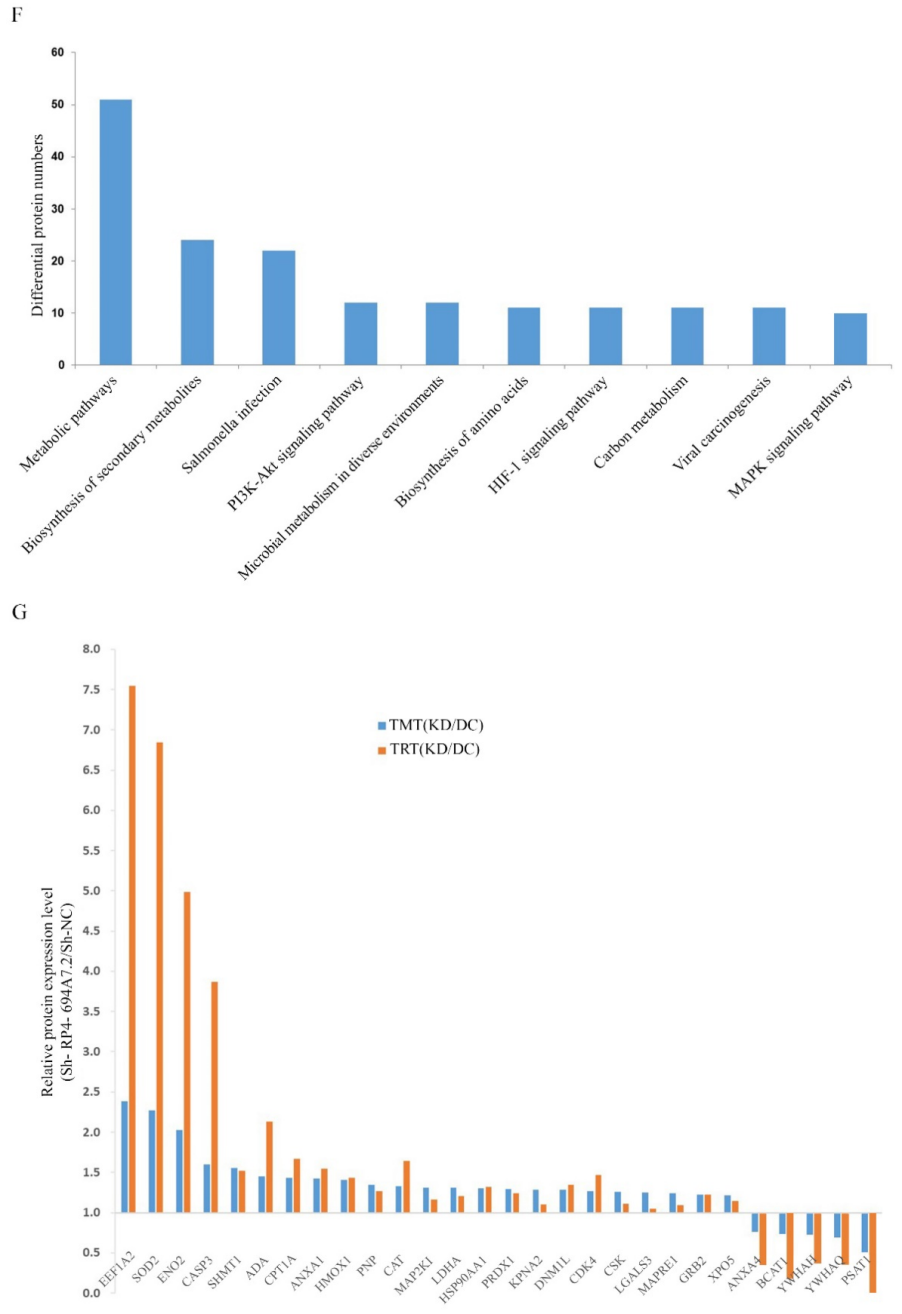
** Screening and identification of differentially expressed proteins associated with RP4-694A7.2. A:** Qualitative and quantitative analyses of proteomics data. Unsupervised hierarchical clustering of all proteome datasets showed separation among the different groups. Protein expression levels are colored, showing higher and lower expression in red and blue, respectively (TMT). **B:** Volcano map of differentially expressed proteins. **C:** Subcellular location of differentially expressed proteins. **D:** FISH assay of the distribution of RP4-694A7.2 expression in cells (×400; red represents RP4-694A7.2 expression and blue represent DAPI nuclear staining). **E:** Gene ontology analysis. The differentially expressed proteins are mainly classified in terms of molecular function, cell composition, and biological process, respectively.** F:** Top 10 pathways of cytoplasmic proteins related to RP4-694A7.2, as determined by KEGG Pathway Analysis. **G:** Comparison of relative expression levels of 28 proteins validated by RPM in SK-Hep-1 cells. Results of the RPM analysis were consistent with TMT results.

To investigate the molecular mechanism underlying the effects of RP4-694A7.2 in HCC, a TMT assay was performed to predict proteins associated with RP4-694A7.2. The TMT assay results are shown in Figure 3A. We identified 1106 proteins that were predicted to interact with RP4-694A7.2 (Figure 3A, 3B). Those proteins were mainly located in the nucleus (29.1%) and cytosol (25.7%) (Figure 3C). Next, a FISH assay was performed using SK-Hep1 cells to detect the location of RP4-694A7.2. Our results showed that RP4-694A7.2 was primarily located in the cytoplasm of SK-Hep1 cells (Figure 3D).

Based on these results, the functional enrichment analysis of 284 cytoplasmic proteins out of 1106 proteins was performed to further investigate their effects, which were mediated by highly expressed proteins associated with RP4-694A7.2, on physiological processes.

The functional protein activities were classified into three major groups by secondary GO analysis: cell component, molecular function, and biological process. Our results showed that cell part (276 proteins), organelle part (227 proteins), and organelle (197 proteins) were the top three cell components where proteins were located. The top three molecular functions were binding (270 proteins), catalytic activity (163 proteins), and molecular function regulation (66 proteins). Regarding biological processes, 264 proteins were related to cellular processes, 233 to biological regulation, and 204 to metabolic processes (Figure 3E).

The differentially expressed plasma proteins associated with RP4-694A7.2 were evaluated by KEGG analysis to analyze the major biological pathways and regulatory processes. The top 10 enrichment analysis pathways are shown in Figure 3F.

### Identification of proteins associated with RP4-694A7.2 in HCC cells

To validate the results of proteomic analyses, we further selected 28 cytoplasmic proteins related to HCC for a PRM analysis. The screening criteria were selected based on the following principles: 1) proteins with potential biological function; 2) fold changes of >1.2 for upregulated proteins and <0.8 for downregulated proteins and a p-value of <0.05 were the established thresholds; and 3) inclusion in the top 10 enrichment analysis pathways.

PRM showed that 23 proteins were upregulated and 5 proteins were downregulated. The PRM results were consistent with those of TMT analysis (Figure 3G). We further selected the proteins whose trend was consistent with that of RP4-694A7.2 for analysis. At last, five proteins-Annexin A4 (ANXA4), tyrosine 3-monooxygenase/tryptophan 5-monooxygenase activation protein theta (YWHAQ), tyrosine 3-monooxygenase/tryptophan 5-monooxygenase activation protein eta (YWHAH), PSAT1, and branched-chain amino acid transaminase 1 (BCAT1)-were included.

### LV-RP4-694A7.2 regulates the progression of HCC by interacting with PSAT1

To further determine whether those proteins (PSAT1, YWHAQ, YWHAH, BCAT1, and ANXA4) mediate the effects of RP4-694A7.2 on proliferation, migration, and invasion in HCC, a phenotypic rescue assay was performed. The results showed that the proliferation of SK-Hep1 cells with RP4-694A7.2 knockdown was significantly reduced, while the phenomenon was partially reversed by PSAT1 and BCAT1 plasmids (p < 0.05). Furthermore, the migration and invasion of SK-Hep1 cells with RP4-694A7.2 knockdown were significantly reduced, while the phenomenon was partially reversed by PSAT1, YWHAQ, YWHAH, and ANXA4 plasmids (p < 0.05).

**Figure 4:**
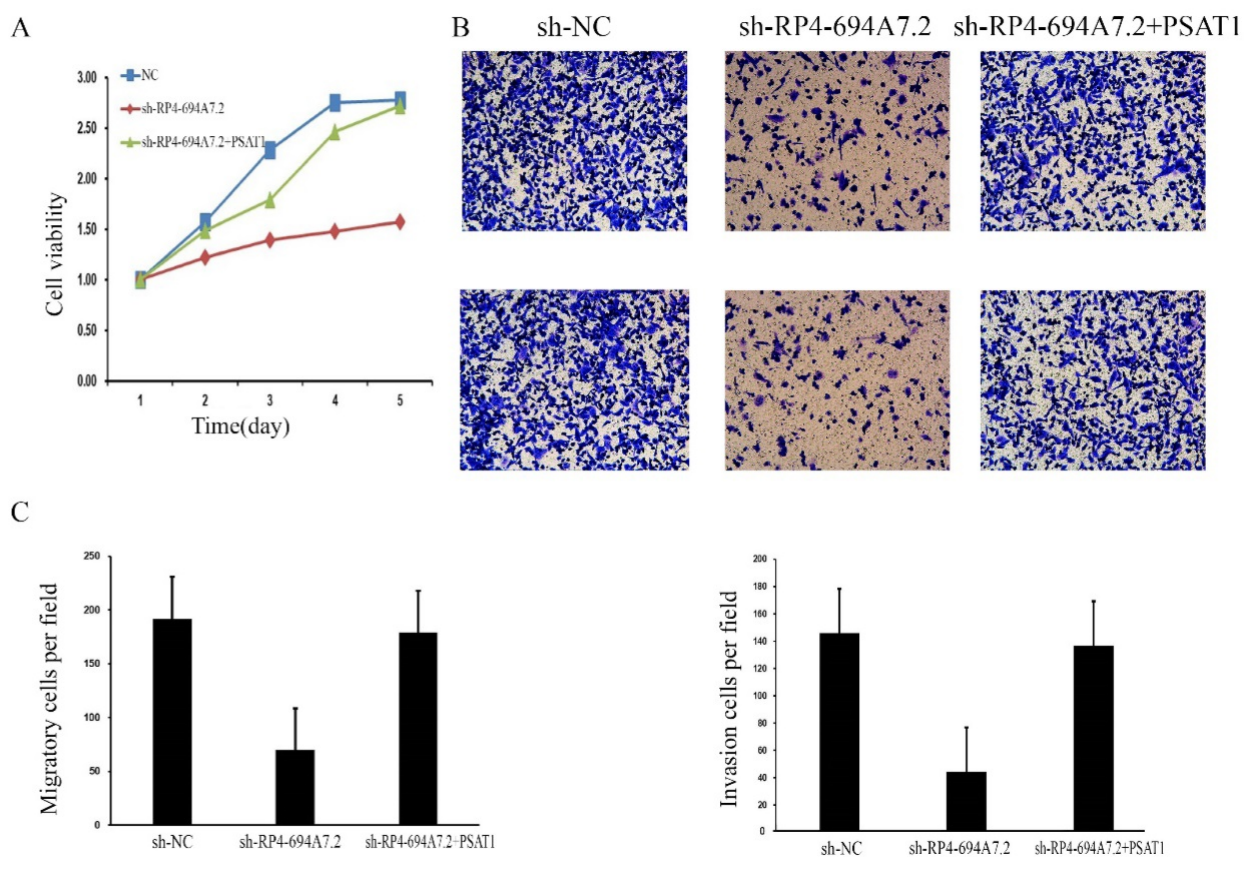
** RP4-694A7.2 promoted cell proliferation, invasion, and migration by regulating PSAT1 in SK-Hep-1 cells. A:** After SK-Hep-1 cells were transfected with sh-NC, sh-RP4-694A7.2, sh-RP4-694A7.2, and PSAT1 plasmids for 5 days, cell proliferation was examined by a CCK-8 assay (p < 0.05). B: After SK-Hep-1 cells were transfected with sh-NC, sh-RP4-694A7.2, sh-RP4-694A7.2, and PSAT1 plasmids for 72 h, their migration and invasion were detected by a Transwell assay. C: Quantification of the number of migration and invasion SK-Hep-1 cells (p < 0.05).

These results showed that PSAT1 is necessary for the RP4-694A7.2-mediated regulation of HCC cell proliferation (Figure 4A), migration, and invasion (Figure 4B).

## Discussion

HCC is a common malignant tumor and its progression is complex, involving cross-talk among many factors, including both genetic and epigenetic changes. Recently, lncRNAs have emerged as important contributors to the biological processes underlying the pathophysiology of many human diseases, including HCC 3. A number of lncRNAs have been identified because of advances in bioinformatics approaches and next-generation sequencing technology. Networks composed of lncRNAs regulate proliferation, metabolism, metastasis, and other processes in various malignant diseases.

In this study, we first identified dysregulated lncRNAs in two GSE data sets related to HCC generated by high-throughput sequencing. We found that RP4-694A7.2 expression is higher in HCC malignant tissues than in normal liver tissues. Recently, RP4-694A7.2 has been found in some solid cancers 14,19; however, its expression patterns and functions in HCC are still unknown. Our results suggested that RP4-694A7.2 is upregulated in HCC and might contribute to tumorigenesis.

Loss-of-function experiments further established the function of RP4-694A7.2 in vitro. The results of these analyses showed that RP4-694A7.2 promotes the proliferation, invasion, and metastasis in SK-Hep1 cells. These results showed that RP4-694A7.2 is an oncogene that promotes HCC development and metastasis. Our results that RP4-694A7.2 is related to increased tumor cell proliferation was consistent with those of a previously published study 19.

To explore the mechanism associated with the effects of RP4-694A7.2, FISH was used to determine the distribution of RP4-694A7.2 in SK-Hep1 cells. Next, TMT was performed to identify the proteins regulated by RP4-694A7.2. As RP4-694A7.2 was mainly distributed in the cytoplasm, 28 cytoplasmic proteins associated with HCC were detected by PRM analysis. Rescue experiments clearly indicated that PSAT1 was overexpressed in HCC and regulated cell proliferation and metastasis.

The serine synthesis pathway (SSP) is an important pathway in cancer metabolism 20. PSAT1, 3-phosphoglycerate dehydrogenase (PHGDH), and phosphoserine phosphatase (PSPH) are three important regulators in the SSP. A study has found that the expression of PSAT1, which is an enzyme that catalyzes the second step of the SSP, is related to cell proliferation and cancer development 21. High PSAT1 expression has been detected in non-small cell lung cancer, breast cancer, and esophageal squamous cell carcinoma, and PSAT1 expression can enhance tumorigenesis and metastasis 22-24. Moreover, PSAT1 could be regarded as a prognostic marker in cancer, as PSAT1 overexpression could indicate a poor prognostic outcome 25. Our results also indicated that PSAT1 is regulated by lncRNA RP4-694A7.2 to modulate HCC cell growth and metastasis.

RP4-694A7.2 has been proven to play an oncogenic role in the cancer development and pathogenesis; it may participate in cell-cycle control, and its silencing reduced the S-phase fraction together with a block of the G1/S phase 19. Our results further showed that numerous proteins that interact with RP4-694A7.2 are associated with catalytic activity and molecular function regulation. PSAT1, a necessary enzyme that makes 3-phosphoglycerate from glycolysis and converts it to serine in the SSP 26, could promote cyclin D1 expression via the GSK3β/β-catenin pathway, which eventually led to the acceleration of cell cycle progression 22. In addition, a previous study showed that PSAT1 may participate in glycolysis and amino acid metabolism in human HCC tissue-derived metabolic 27. Consistent with our PRM and TMT results, metabolic pathways were the most enriched. In summary, we speculated that RP4-694A7.2 may regulate glycolysis function by PSAT1 via the GSK3β/β-catenin pathway during HCC cell growth and metastasis.

This study also has several limitations. Although we identified that RP4 694A7.2 may act as an oncogene to promote cell proliferation, invasion, and migration by targeting PSAT1, more regulatory factors and molecular elements that interact are still unknown. Further studies are required to clarify these molecular elements and the interactions among them. In addition, the clinical application of RP4 694A7.2 needs to be explored in the future.

In summary, the results of our study illustrate that RP4-694A7.2 is upregulated in HCC and promotes cell proliferation and metastasis via PSAT1.

## Figures and Tables

**Table 1:** Primers used in lncRNA screening

Gene Name		Sequence
CTB-147C22.8	-forward	5'- CTCAGGTAGAGAGGAACCACAAGG-3'
	- reverse	5'- AGGCAGGGAAGGAGAGGTGT -3'
RP4-694A7.2	-forward	5'- GTGATGTAGCCACAAACAACTACCA -3'
	- reverse	5'- GGGAAAACCACTCATCTGTCTTG -3'
RP11-428C19.4	-forward	5'-ACAGTCTGGAATTTACGAGGATGAG -3'
	- reverse	5'-CCTGTGTGTGGTTGACTTCTTTTG -3'
HAO2-IT1	-forward	5'-TTTGCCTTCTTGAGGATGGAG-3'
	- reverse	5'-GGAGTGGGAATAGCAGGTGTG -3'
HOXD-AS2	-forward	5'-CATAGCCATGCAGCCTTCAG-3'
	- reverse	5'-AGTGCTATTTCACATCCAAGCTTCT-3'
HOXA10-AS	-forward	5'-CCGCAAATGCGTCAAGGA-3'
	- reverse	5'-AAGGGCTTACAGGCATGGACA-3'
GAPDH	-forward	5'-GCACCGTCAAGGCTGAGAAC-3'
	- reverse	5'-TGGTGAAGACGCCAGTGGA-3'
